# The relationship between the type of unstable intertrochanteric femur fracture and mobility in the elderly

**DOI:** 10.1186/s13018-018-0911-1

**Published:** 2018-08-22

**Authors:** O. Karakus, G. Ozdemir, S. Karaca, M. Cetin, B. Saygi

**Affiliations:** 1 Fatih Sultan Mehmet Training and Research Hospital, Omer HalisDemir University Hospital, Petrolıs st. Sümer bloc no: A-16 Kartal, İstanbul, Turkey; 2 Fatih Sultan Mehmet Training and Research Hospital, Istanbul, Turkey; 3 Ankara Numune Training and Research Hospital, Ankara, Turkey; 4 Maltepe University, Istanbul, Turkey

**Keywords:** Intramedullary nailing, Intertrochanteric fractures, Fracture fixation, Early mobilization

## Abstract

**Background:**

The purpose of this study was to assess the impact of uniform anti-rotational proximal femoral intramedullary nail (APFN) use on patient mobility status with the treatment of two different unstable intertrochanteric femur fracture groups of geriatric patients.

**Methods:**

The study included patients aged > 65 years who underwent surgery with APFN. Group 1 comprised AO classification, AO/OTA 31-A22, and A23 patients, and group 2, A31 and A32 patients. The demographic data of the patients, postoperative complications, follow-up, mortality status, postoperative reduction, tip-apex distance (TAD), and the Parker-Palmer mobility (PPM) score were evaluated.

**Results:**

There were no statistically significant differences between the groups in terms of gender, affected side, time from trauma to surgery, ASA score, anesthesia type, duration of hospitalization, duration of surgery, TAD values, reduction values, or mortality rate. The average age of patients in group 2 was significantly higher than that of patients in group 1 (*p* < 0.05). The mobility scores of group 1 patients were significantly higher than those of group 2 (*p* < 0.05).

**Conclusions:**

While no relationship was found between the TAD values and the reduction status of the cases, the PPM scores of the AO 31 A3 cases were determined to be significantly worse. Therefore, fractures with a preoperative classification of AO type 31 A3 can be expected to have worse results than A2 ITF fractures. The fracture type seems to have as great an effect as other factors on the postoperative mobility score.

## Background

With increasing average life expectancy, there is a parallel increase in the elderly population and this has also been shown to have increased the incidence of geriatric fractures, with a consequent significant increase in mortality and economic costs [1, 2]. Unstable fracture patterns are known to occur more often with decreased bone mineral density and advanced age [3].

Early mobilization and full weight-bearing of the patient are recommended to prevent complications of immobilization after intertrochanteric fracture (ITF) surgery. Although advantages and disadvantages have been demonstrated of each dynamic hip screw and intramedullary nailing method commonly used in the treatment of these fractures, the most common cause of mechanical failure of these methods is cut-out in the hip screw [4]. Therefore, as intramedullary nails permit early weight-bearing, this method is more advantageous in reducing complications, particularly in unstable ITF [5].

The pre-fracture functional level, age, and fracture type have been reported to be predictors of patient mobility in the elderly [6, 7]. However, previous studies evaluating the effect of fracture type on patient mobility have specifically investigated intertrochanteric and cervical fractures [8]. In our clinical practice, some differences have been observed in mobility patterns following the treatment of unstable ITF. To the best of our knowledge, there has been no study in literature that has compared unstable ITF patterns in terms of mobility status.

The purpose of this study was to assess the impact of the use of uniform anti-rotational proximal femoral intramedullary nail (APFN) on patient mobility with the treatment of two different unstable ITF groups of geriatric patients.

## Methods

The study included patients aged > 65 years who underwent surgery for unstable ITF using APFN with a minimum 12-month follow-up period. The unstable fractures were divided into two groups according to the Arbeitsgemeinschaft für Osteosynthesefragen (AO) classification. Group 1 comprised AO/OTA 31-A22 and A23 fractures and group 2, A31 and A32 [9]. All patients provided written informed consent prior to inclusion in the study.

Patients with no regular postoperative follow-up visits, open or pathological fractures, a previous proximal femoral fracture, or additional fractures preventing mobility, and those with poor quality radiographs were excluded from the study.

### Surgical procedure

In all cases, the operation was performed with the patient in a supine position on a traction table after closed reduction under fluoroscopic control. Osteosynthesis was applied with A-PFN® (TST, Istanbul, Turkey) nail of 220 mm length, 15 mm proximal diameter, and a lateral angle of 6° in the proximal section. It also has a distal slit to reduce stress and a lag screw of 10 mm diameter applied with the antirotator blade. All patients were administered 4 × 1 gr cefazolin sodium intravenously as prophylactic at 24 h postoperatively. For thromboembolism prophylaxis, enoxaparin was administered to each patient according to the weight and risk of hospitalization and was continued for 14 days postoperatively. On postoperative day 1, quadriceps exercises were started and all patients were mobilized with a walker and weight-bearing as tolerated.

### Clinical data

The clinical data of the patients were obtained from hospital records. The demographic data of the patients, fractured side, time from trauma to surgery, the American Society of Anaesthesiologists (ASA) score, operating time, postoperative complications, follow-up examinations, and mortality status were recorded for each patient.

The mobility status of the patients was evaluated at the final follow-up examination and was scored between 0 and 9 according to the Parker-Palmer mobility (PPM) score [6, 10]. Fracture classification, position of the screw, and tip-apex distance (TAD) were evaluated on anteroposterior and lateral radiographs in the PACS (Picture Archiving and Communication System) system or in the patient file. The Baumgaertner criteria, modified by Fogagnolo, were used for the evaluation of the reduction postoperatively [11]. All the measurements were performed on early postoperative radiographs.

### Statistical analysis

Data obtained in the study were statistically analyzed using NCSS (Number Cruncher Statistical System) 2007 Statistical Software (Utah, USA). Descriptive statistical methods (mean, standard deviation, median, frequency, rate, minimum, maximum) and quantitative data methods were used with Student’s *t* test applied for the comparison of two groups of variables with normal distribution, and the Mann-Whitney *U* test was applied when distribution was not normal. In the comparison of qualitative data, the Fisher’s exact test, Fisher-Freeman-Halton test, and Yates Continuity Correction test (Yates Chi-square) were used. A value of *p* < 0.05 was considered statistically significant.

## Results

Within the specified study period, a total of 164 geriatric cases were operated on for unstable ITF. Of these, 110 cases were excluded due to a lack of regular follow-up for 12 months postoperatively or poor quality radiographs. Thus, the study was conducted with a total of 54 cases, comprising 29 in group 1 and 25 in group 2. The demographic characteristics of the patients are shown in Tables 1 and 2.

**Table 1:** Demographic characteristics I

	Min-Max	Mean ± SD
Age	65–94	79.28 ± 9.54
Hospitalization time	4–18	8.89 ± 2.89
Waiting time	3–17	7.85 ± 2.83
Operation time	30–90	55.19 ± 15.51

**Table 2:** Demographic characteristics II

	*N*	%
Sexuality		
Female	43	79.6
Male	11	20.4
Side		
Left	24	44.4
Right	30	55.6
ASA		
2	10	18.5
3	39	72.2
4	5	9.3
Anesthesia		
General	9	16.7
Spinal	45	83.3

Of the patients included in the study, 43 were female and 11 were male with a mean age of 79.28 ± 9.54 years (range 65–94 years). The operated side was the left side in 24 cases and the right side in 30.

The ASA score was recorded as 2 in 10 cases, 3 in 39 patients, and 4 in 5 cases. General anesthesia was applied to 16.7% of the patients, and spinal anesthesia to 83.3%.

The average time from trauma to surgery was 7.85 ± 2.83 days (range 3–17 days), the average operation time was 55.19 ± 15.51 min (range 30–90), and the average length of stay in hospital was 8.89 ± 2.89 days (range 4–18 days).

The distribution of cases according to AO classification are shown in Table 3; 24 were classified as 31A22, 5 cases as 31A23, 18 cases as 31A31, and 7 cases as 31A32.

**Table 3:** Fracture type distribution of patients according to AO classification

	*N*	%
AO classification		
2-2	24	44.4
2-3	5	9.3
3-1	18	33.3
3-2	7	13.0

Postoperatively, the average TAD value of the cases was 17.11 ± 5.46 mm (range 5–30 mm) and the mean PPM mobility score was 4.54 ± 3.54 (range 0–9). The reduction values were good in 61.1% of all the cases, average in 16.7%, and poor in 22.2%.

During the follow-up period, mortality was observed in 13 cases, comprising 8 cases in group 1 and 5 from group 2. Revision surgery was necessary in 3 patients, 1 in group 1 and 2 in group 2, due to cut-out complications.

The mean follow-up time was 15.23 months (range 12–22 months) in group 1 and 12.95 months (range 12–18 months) in group 2. PPM mobility scores were measured in the final follow-up examination as 5.62 ± 3.41 and 3.28 ± 3.32 in group 1 and group 2 respectively.

The evaluations according to the groups are shown in Tables 4 and 5. A statistically significant difference was found between the groups in respect of age (*p* = 0.033; *p* < 0.05) with the average age of patients in group 2 significantly higher than that of group 1 patients.

**Table 4:** The distribution of descriptive characteristics according to the groups-I

	Group 1 (*n* = 29)	Group 2 (*n* = 25)	*p*
	Mean ± Sd	Mean ± Sd	
Age	76.79 ± 10.70	82.16 ± 7.17	0.033^a^
Waiting time	7.76 ± 3.18	7.96 ± 2.42	0.797^a^
Hospitalization time	8.79 ± 3.27	9 ± 2.43	0.796^a^
Operation time	56.21 ± 15.91	54 ± 15.28	0.607^a^
TAD	16.79 ± 5.16	17.48 ± 5.88	0.649^a^
Mobility (median)	5.62 ± 3.41 (7)	3.28 ± 3.32 (2)	0.019^b^
	*n* (%)	*n* (%)	*p*
Sexuality			
Female	23 (79.3)	20 (80.0)	1.000^c^
Male	6 (20.7)	5 (20.0)	
Side			
Right	15 (51.7)	9 (36.0)	0.376^c^
Left	14 (48.3)	16 (64.0)	

^a^Student *t* test

^b^Mann Whitney *U* test

^c^Yates Continuity Correction test

**Table 5:** The distribution of descriptive characteristics according to the groups

	Group 1 (*n* = 29)	Group 2 (*n* = 25)	*p*
	*n* (%)	*n* (%)	
ASA			
2	5 (17.2)	5 (20)	0.743^d^
3	22 (75.9)	17 (68)	
4	2 (6.9)	3 (12)	
Anesthesia			
General	3 (10.3)	6 (24)	0.275^e^
Spinal	26 (89.7)	19 (76)	
Reduction			
Good	19 (65.5)	14 (56.0)	0.687^d^
Poor	5 (17.2)	4 (16.0)	
Moderate	5 (17.2)	7 (28.0)	
Mortality			
Exitus	8 (27.6)	5 (20.0)	0.741^c^
Alive	21 (72.4)	20 (80.0)	
AO classification			
2-2	24 (82.8)	0 (0.0)	0.001^d**^
2-3	5 (17.2)	0 (0.0)	
3-1	0 (0.0)	18 (72.0)	
3-2	0 (0,0)	7 (28.0)	

^c^Yates Continuity Correction test

^d^Fisher-Freeman-Halton test (Monte Carlo)

^e^Fisher’s Freeman tests

^**^p < 0.01

A statistically significant difference was detected between the groups in respect of the mobility scores of patients (*p* = 0.019; *p* < 0.05). The mobility scores of group 1 patients were determined to be significantly higher than those of group 2 patients.

There were no statistically significant differences between the groups in terms of gender, operated side, time from trauma to surgery, ASA score, anesthesia type, duration of hospitalization, duration of surgery, TAD values, reduction values, and mortality rate.

## Discussion

As early mobilization and re-gaining the pre-fracture level of function in the shortest possible time are of critical importance in the treatment of hip fractures [12], cases with ITF should be mobilized as soon as possible to prevent the emergence of complications that could increase mortality. Early ambulation has been shown to be one of the most effective ways of reducing mortality [13, 14]. Therefore, surgical treatment is the first choice in patients with ITF. While some authors claim that surgical treatment should be applied in the first 48 h if possible, others have stated that it should only be applied after the patient has become stable internally [15]. In the current study, it was only possible to operate at an average of 7.85 days after admission, because of systemic problems and the high volume of patients in the hospital.

Fragmentation of the posteromedial cortex, subtrochanteric extension, and reverse oblique fracture line are major causes of instability. Studies comparing intramedullary implants with extramedullary implants have revealed similar success rates for stable fractures, whereas intramedullary options have been shown to have higher success rates and lower complication rates in unstable fractures [16, 17]. High complication rates have been reported in the osteosynthesis of these fractures with extramedullary implants [18]. In general, 14 times more cut-outs are observed in these cases and this can be explained by the fractures being unstable, and therefore, reduction is difficult [19]. It has also been reported that the use of intramedullary nails in the treatment of ITF is increasing [20]. In the current study, APFN was selected for use in patients with unstable fractures.

Tip-apex distance (TAD) is an effective indicator to determine the possibility of cut-out of the screw from the femoral head. Screws with TAD > 25 mm are particularly at risk of cut-out. The surgeon’s attention to TAD reduces the risk of femoral head cut-out of the screw [21]. In a study by Pervez et al., cut-out was not observed in any patient with TAD < 25 mm, whereas 2% of patients with TAD 25–30 mm and 27% of patients with TAD > 30 mm showed peeling. In another study, it was claimed that TAD should be < 20 mm [22]. In the present study, the average TAD of the patients was measured as 16.79 mm in group 1 and 17.48 mm in group 2, with no statistically significant difference determined between the two groups (*p* > 0.05).

There are several studies in literature related to the complications that can develop postoperatively, such as non-union, delayed union, implant failure, proximal screw cut-out, and Z-effect [23]. In the current study, cut-out was seen in 1 patient of group 1 and in 2 patients of group 2. Furthermore, mortality developed during the follow-up period in a total of 13 cases, as 8 in group 1 and 5 in group 2.

The position of the screw in the femoral head is another important factor, and in the current study, the center-inferior position was preferred, as recommended in literature [24].

The 1-year mortality rate after unstable ITF varies between 11 and 27% in literature [25]. In the current study, this mortality rate was 27.6% for A22-A23 patients and 20% for A31-A32, with no statistically significant difference determined between the groups.

It has been reported that indications for using standard or long intramedullary nails in the treatment of unstable ITF are unclear and are almost subjective [26]. The selection of long intramedullary nails to reduce re-operation and non-union rates is also under question. It has been suggested that reverse oblique ITF can be treated with both standard and long intramedullary nails. In a study by Okcu et al. comparing the results of the use of long and standard PFN, the average PPM score of the cases in the standard PFN group was reported to be 5.5. Ellis stated that the mean PPM score was 5 in cases where expandable PFN had been applied [25–28].

In the present study, with the use of short intramedullary nails (APFN®), the average PPM scores of patients with type 31 A2 and A3 fractures were 5.62 and 3.28 respectively and there was a statistically significant difference between these fracture groups (*p* = 0.019; *p* < 0.05). These results obtained with short PFN in unstable fractures can be considered good and satisfactory compared with the findings in literature.

While no relationship was found between the TAD value and reduction status, the PPM scores of the AO 31 A3 cases were significantly worse. Therefore, it can be said that the mobilization status of patients with an A3 fracture is worse despite undergoing surgery of the same quality. However, these A3 patients were both older and had a more unstable fracture, thereby resulting in worse mobility scores. Thus, it can be preoperatively anticipated that AO type 31 A3 fractures will have worse outcomes than A2 fractures.

A statistically significant difference was determined between the study groups in respect of age, with the mean age of group 2 patients significantly higher than that of patients in group 1. In addition, the mobility scores of group 1 were significantly higher than those of group 2, so it was seen that the mobility score decreased with increasing age.

Limitations of the current study could be said to be the small number of the case group and an insufficient follow-up period for some cases due to mortality.

In the light of the results of the present study, it can be concluded that satisfactory results can be obtained with the use of anti-rotational proximal femoral nail in the treatment of geriatric unstable ITF. It seems clear that the fracture type is more predictive of the postoperative mobilization status of the patients than other factors.

## Conclusions

While no relationship was found between the TAD value and reduction status of the cases, the PPM scores of the AO 31 A3 cases were significantly worse. Therefore, it can be preoperatively anticipated that AO type 31 A3 fractures will have worse outcomes than A2 fractures and it is clear that the fracture type affects the mobility score at least as much as other factors.

## Abbreviations

AO: Arbeitsgemeinschaft für Osteosynthesefragen
APFN: Antirotational proximal femoral intramedullary nail
ASA: American Society of Anaesthesiologists
ITF: Intertrochanteric fractures
NCSS: Number Cruncher Statistical System
PACS: Picture Archiving and Communication System
PPM: Parker-Palmer mobility
TAD: Tip-apex distance
